# Cold Temperature Delays Wound Healing in Postharvest Sugarbeet Roots

**DOI:** 10.3389/fpls.2016.00499

**Published:** 2016-04-14

**Authors:** Karen K. Fugate, Wellington S. Ribeiro, Edward C. Lulai, Edward L. Deckard, Fernando L. Finger

**Affiliations:** ^1^Northern Crop Science Laboratory, United States Department of Agriculture-Agricultural Research ServiceFargo, ND, USA; ^2^Departamento de Fitotecnia, Universidade Federal de ViçosaViçosa, Brazil; ^3^Department of Plant Sciences, North Dakota State UniversityFargo, ND, USA

**Keywords:** *Beta vulgaris*, harvest injury, lignin, respiration, storage, suberin

## Abstract

Storage temperature affects the rate and extent of wound-healing in a number of root and tuber crops. The effect of storage temperature on wound-healing in sugarbeet (*Beta vulgaris* L.) roots, however, is largely unknown. Wound-healing of sugarbeet roots was investigated using surface-abraded roots stored at 6 and 12°C for 28 days. Surface abrasions are common injuries of stored roots, and the storage temperatures used are typical of freshly harvested or rapidly cooled roots. Transpiration rate from the wounded surface and root weight loss were used to quantify wound healing. At 12°C, transpiration rate from the wounded surface declined within 14 days and wounded roots lost weight at a rate similar to unwounded controls. At 6°C, transpiration rate from the wounded surface did not decline in the 28 days after injury, and wounded roots lost 44% more weight than controls after 28 days storage. Melanin formation, lignification, and suberization occurred more rapidly at 12°C than at 6°C, and a continuous layer of lignified and suberized cells developed at 12°C, but not at 6°C. Examination of enzyme activities involved in melanin, lignin, and suberin formation indicated that differences in melanin formation at 6 and 12°C were related to differences in polyphenol oxidase activity, although no relationships between suberin or lignin formation and phenylalanine ammonia lyase or peroxidase activity were evident. Wound-induced respiration was initially greater at 12°C than at 6°C. However, with continued storage, respiration rate of wounded roots declined more rapidly at 12°C, and over 28 days, the increase in respiration due to injury was 52% greater in roots stored at 6°C than in roots stored at 12°C. The data indicate that storage at 6°C severely slowed and impaired wound-healing of surface-abraded sugarbeet roots relative to roots stored at 12°C and suggest that postharvest losses may be accelerated if freshly harvested roots are cooled too quickly.

## Introduction

At the end of the production season, leaves are removed from sugarbeet (*Beta vulgaris* L.) roots with hard rubber flails that beat the leaves off of the subtending taproot or with knives that slice off the shoot and a small portion of the upper taproot. Roots are mechanically lifted from the soil, bounced aggressively over chains or rollers to remove dirt, and dropped into a truck. Roots are then transported to piling stations, dumped from trucks into a sugarbeet piler, and dropped again as the roots are piled. As a consequence of these harvest and piling operations, sugarbeet roots placed into storage sustain extensive injuries (Parks and Peterson, 1979; Bentini et al., 2002). A study with conventionally harvested and piled roots showed that of the roots placed into storage, 89% were bruised, 58% had the lower tail portion of the root broken off, and 38% were cracked (Steensen, 1996). In addition to bruises, cracks, and root breakage, sugarbeet roots in storage commonly have cuts, surface abrasions, and loss of small fragments of the root (Wiltshire and Cobb, 2000).

Healing of wounds incurred during harvest and piling operations is necessary to reduce dehydration and microbial infection of sugarbeet roots during storage. Dehydration causes storage and processing losses since it accelerates respiration rate, sucrose loss, and the formation of glucose and fructose, two compounds that impede sugarbeet root processing and increase sucrose loss to molasses in the factory (Dexter et al., 1969; Lafta and Fugate, 2009). Unhealed wound sites provide opportunistic plant pathogens access to internal plant tissue and increase the incidence of storage diseases. Without injury, two of the most common storage rot pathogens of sugarbeet, *Penicillium* spp. and *Botrytis cinerea*, are unable to establish infection (Mumford and Wyse, 1976).

In other root and tuber crops, including potato, sweet potato, yam, carrot, and cassava, storage temperature affects the rate and extent of wound healing (Nielsen and Johnson, 1974; Wiggington, 1974; Garrod et al., 1982; Diop, 1998). As a consequence, these crops, with the exception of carrot, are typically ‘cured’ after harvest by storing them for a short period, typically 4–14 days, at temperatures well in excess of the temperatures at which they are held for long-term storage to permit harvest-incurred wounds to heal. For sugarbeet roots destined for storage, no curing period at elevated temperatures is employed. Roots are harvested in late autumn after soil temperatures have declined and root internal temperatures are typically below 13°C (Campbell and Klotz, 2006; Bernhardson, 2009). Once piled, roots are cooled as rapidly as possible using the cool ambient air available in late autumn and early winter.

Knowledge of wound-healing events and their interaction with storage temperature is limited for sugarbeet roots. Previously, Ibrahim et al. (2001) described the microscopically detectable wound-healing processes (i.e., suberization, lignification, and melanin formation) that occurred in bruised sugarbeet roots stored at 10°C, and Artschwager and Starrett (1933) investigated the effect of temperature and humidity on suberin formation in cut roots. Here, we describe the effect of temperature on wound healing in surface-abraded sugarbeet roots stored at 12 or 6°C, two temperatures that are typical of freshly harvested roots or roots that have been subjected to cooling. Root transpiration rate, root weight loss, suberin formation, lignin formation, and melanin synthesis were determined as indicators of wound healing, and the underlying mechanisms for differences in wound-healing were investigated by determining relationships between wound-healing processes and the enzyme activities that contribute to them. The purpose of this research was to generate information to improve our understanding of the effect of temperature on sugarbeet root wound-healing processes which will aid in understanding storage losses and the effect of current storage practices on these losses.

## Materials and Methods

### Plant Material, Treatments, and Storage Conditions

Sugarbeet plants of hybrid VDH66156 (SESVanderhave, Tienen, Belgium) were greenhouse-grown in Sunshine Mix #1 (Sun Gro Horticulture, Vancouver, BC, Canada) in 15 L pots with supplemental light using a 16 h light/8 h dark regime. Plants were watered as needed and fertilized with a controlled release fertilizer (Multicote 4, Sun Gro Horticulture). Roots were harvested 16–18 weeks after planting. Leaves and vegetative buds were removed with a knife, and roots were washed to remove potting media. Roots were randomized. Half of the roots were wounded by scraping the root surface 2–3 times with a steel brush 2 h after harvest. The remaining roots served as unwounded controls. Roots were placed in perforated, plastic, 5 kg potato bags, approximately 5 roots per bag, for storage. Half of the wounded roots and half of the control roots were stored at 6°C for up to 4 weeks. The remaining roots were stored at 12°C for up to 4 weeks. Relativity humidity inside bags was 94%. Whole roots were used to determine respiration rate and weight loss, with eight replicates per treatment, using the same roots for all time points. Tissue cylinders (15 mm × 20 mm, diameter × height) that contained the injured or uninjured surface of the root were obtained from wounded or control roots, respectively, and were used for transpiration determinations, using five replicate roots per treatment. Tissue cylinders were obtained from the widest portion of the root with a cork borer. Tissue samples collected from wounded and control roots 0.5 h after injury and after 7, 14, 21, and 28 days in storage were used to determine enzymatic activities, total phenolic concentration, and antioxidant capacity, with five replicate roots per treatment. Tissue samples were collected from the outer surface of roots, at the widest portion of the root, using a potato peeler. These samples, containing approximately 2 mm of the outermost root tissue, were flash frozen in liquid nitrogen, freeze-dried, ground to a fine powder, and stored at -80°C prior to analysis. Tissue blocks (approximately 1 cm^3^) that contained the outermost cell layers of wounded and control roots were obtained with a razor blade and used for microscopy. Samples were collected from the widest part of roots 0.5 h after injury and after 7, 14, 21, and 28 days in storage.

### Transpiration and Respiration Rate Determinations

Tissue cylinders that contained the injured or uninjured surface of wounded and unwounded roots, respectively, were collected as described above. All sides of the cylinders except the root surface were wrapped with DuraSeal (Diversified Biotech, Boston, MA, USA), and the film-covered portions of the cylinder were inserted into a test tube (15 mm diameter) to ensure that transpiration was only from wounded or unwounded root surfaces. Transpiration rate was determined by the linear regression of weight loss versus time using 5 hourly measurements of weight loss.

Respiration rates of roots were determined after 1, 2, 3, 4, 5, 6, 7, 14, 21, and 28 days in storage. The CO_2_ produced by individual roots was measured by infrared gas analysis using an open system comprised of a LI-COR 6400 gas analyzer (Lincoln, NE, USA) attached to a 7 L sample chamber and maintained with a 1000 μmol s^-1^ flow of air (Haagenson et al., 2006).

### Microscopy and Histology

Tissue blocks that contained the injured surface of wounded roots or the uninjured surface of control roots were hand-sectioned with a razor blade. Autofluorescence was used to detect polyphenolics in lignin and suberin. Samples were illuminated with UV epifluorescent light from an HBO 50 W (L2) mercury short arc lamp equipped with a G-365 exciter filter, a FT-395 chromatic beam splitter, and a LP-429 barrier filter (Sabba and Lulai, 2002). Images were captured using a Zeiss Axioskop 50 microscope (Jena, Germany) and Zeiss color AxioCam camera. To detect lignin, sections were stained with saturated phloroglucinol in 5 N HCl and examined using standard light microscopy (Jensen, 1962).

### Enzyme Activity Assays

Peroxidase (POD), polyphenol oxidase (PPO), phenylalanine ammonia lyase (PAL), and catalase (CAT) activities were determined using the protein extraction and activity assay methods of Aebi (1984), Campos et al. (2004), Wuyts et al. (2006), and Ferrareze et al. (2013), respectively. Protein extracts were prepared by adding five volumes (w/v) of an extraction buffer to freeze-dried tissue. Extraction buffers were for POD: 0.1 M potassium phosphate buffer, pH 7.0, 10 mM sodium bisulfite, and 0.5 M NaCl; for PPO: 0.1 M potassium phosphate buffer, pH 6.5, and 1% polyvinylpyrrolidone-40 (PVP-40); for PAL: 0.1 M Tris-HCl, pH 7.2, 2 mM sodium bisulfite, 5 mM 2-mercapethanol, and 2.5% insoluble polyvinylpolypyrrolidone; for CAT: 0.1 M potassium phosphate, pH 7.0, 1 mM EDTA, 1 mM PMSF, and 1% (w/v) PVP. Suspensions of tissue and extraction buffer were sonicated for 15 min at 4°C, filtered over Miracloth (EMD Millipore, Billerica, MA, USA), and centrifuged at 16,000 *g* for 15 min at 4°C. Supernatants containing the soluble protein extracts were used for enzyme activity and total soluble protein assays.

Enzyme activities were measured spectrophotometrically using a Shimadzu model UV-1601 dual beam spectrophotometer (Kyota, Japan). POD activity assays contained protein extract, 0.1 M potassium phosphate buffer, pH 6.5, 10 mM guaiacol, and 4 mM hydrogen peroxide. Activity was determined at 25°C using the maximum change in absorbance at 470 nm during the first 3 min of the reaction and an extinction coefficient of 26.6 mM^-1^ cm^-1^ (Koduri and Tien, 1995). PPO activity assays contained protein extract, 0.1 M phosphate buffer, pH 6.5, and 50 mM catechol. Activity was determined at 25°C using the maximum change in absorbance at 420 nm. PAL activity assays contained protein extract, 0.1 M HEPES, pH 8.0, and 10 mM *l*-phenylalanine. Reactions were incubated at 40°C for 0.5 h and terminated by addition of trichloroacetic acid to a final concentration of 10%. Terminated reactions were incubated for 5 min at room temperature and centrifuged for 10 min at 16,000 *g*. Activity was determined by the change in absorbance at 290 nm against a cinnamic acid standard curve. CAT activity assays contained protein extract, 50 mM phosphate buffer, pH 7.0, and 10 mM H_2_O_2_. Activity was determined at 25°C using the change in absorbance at 240 nm and an extinction coefficient of 39.4 mM^-1^ cm^-1^(Koduri and Tien, 1995). Total soluble protein concentrations were determined using Bio-Rad Protein Assay Reagent (Hercules, CA, USA) with bovine serum albumin as a standard.

### Soluble Phenolic Compounds and Antioxidant Capacity

Extracts of soluble phenolic compounds were prepared by adding 1 mL 50:3.7:46.3 (v/v) solution of methanol:acetic acid:water to 15 mg freeze-dried tissue, sonicating for 15 min, and centrifuging at 16,000 *g* for 15 min. An aliquot of the extract (0.2 mL) was added to a 1:10 (v/v) solution of Folin-Ciocalteu reagent:water (1 mL), and incubated for 10 min at room temperature (Fu et al., 2010). A 7.5% solution of sodium carbonate (0.8 mL) was added, and the resulting solution was mixed and incubated for 0.5 h at room temperature. Soluble phenolic compound concentrations were determined by absorbance at 473 nm using gallic acid as a standard.

Extracts for antioxidant capacity determinations were prepared by adding 50 volumes (v/w) of 70% methanol to freeze-dried tissues. Solutions were vortexed, sonicated for 15 min, filtered over Miracloth, and centrifuged at 16,000 *g* for 15 min. Aliquots of supernatants were added to a 1:2 solution of 0.5 mM ethanolic α,α-diphenyl-β-picrylhydrazyl (DPPH):100 mM sodium acetate, pH 5.5 (Kang and Saltveit, 2002). Solutions were vortexed for 13 s and allowed to stand for 0.5 h. Antioxidant capacity was determined by the change in absorbance at 517 nm.

### Statistical Analysis

Statistical analyses were conducted with Minitab Statistical Software (ver. 16.2.3, State College, PA, USA). Analysis of variance and Tukey range tests were used to determine significant differences between wounding treatments, storage temperatures, and time points. Results of ANOVAs are available in Supplementary Table S1. Relationships between measured parameters were determined using Pearson product-moment correlations. For all analyses, α = 0.05.

## Results

### Transpiration and Dehydration

**FIGURE 1:**
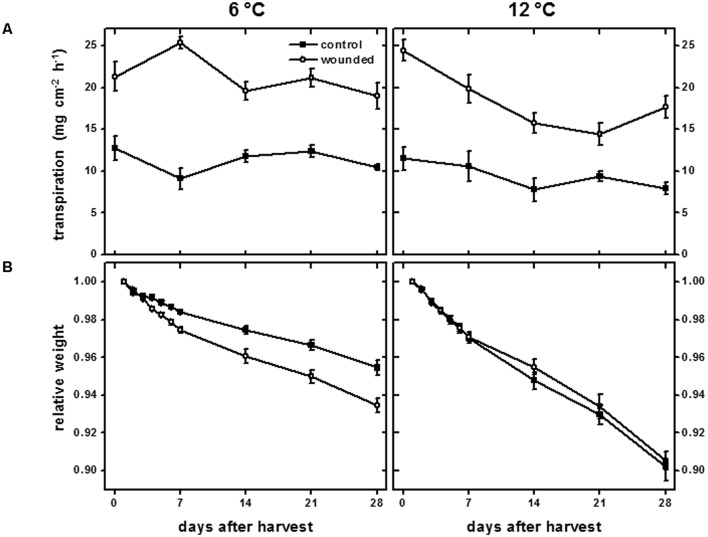
**Transpiration rate (A) of wounded and unwounded (control) tissues and relative weight (B) of wounded and unwounded (control) roots during 28 days storage at 6 and 12°C.** Roots were wounded by abrading the root surface with a wire brush 2 h after harvest and were subsequently stored at 94% relative humidity. Transpiration rate was measured as the rate of water lost from tissue disks from the surface of wounded or control roots. Relative weight was the weight of roots relative to their weight on the day of harvest after wounding. Data are the mean ± SE of the mean. For transpiration rate, *n* = 5; for relative weight, *n* = 8.

The transpiration rate of wounded tissue and the weight loss of plant organs are common measures of wound-healing (Bajji et al., 2007; Lulai et al., 2008) and were used to evaluate the wound-healing of surface-abraded sugarbeet roots that were stored at 6 or 12°C for 28 days (**Figure 1**). Wounding increased the transpiration rate from the surface of roots at both 6 and 12°C throughout the entire 28 days of the experiment (**Figure 1A**). At 12°C, the transpiration rate of wounded tissue declined with time in storage and was significantly reduced 14 days after harvest. The decrease in transpiration rate indicated a reduction in the permeability of the wounded surface to water and healing of the injury (Jarvis and Duncan, 1979). No significant decline in transpiration rate was observed for wounded tissue from roots stored at 6°C. At this temperature, roots were unable to restrict water loss during 28 days storage, presumably due to a lack of wound-healing.

Weight loss from roots stored at 6 and 12°C substantiated the occurrence of wound healing at 12°C, but not at 6°C (**Figure 1B**). Weight loss in storage is primarily due to water loss (Akeson et al., 1974), and the extent that wounded roots maintained weight served as a measure of the root’s ability to seal off wound sites and limit desiccation. At 12°C, wounded and unwounded roots lost weight at similar rates. Dehydration, therefore, was not accelerated in wounded roots at 12°C relative to unwounded controls, presumably due to healing of the abraded surface. At 6°C, wounded roots lost weight more rapidly than unwounded controls, indicating a substantial reduction in wound-healing compared to roots stored at 12°C. After 28 days, wounded roots stored at 6°C lost 44% more weight than uninjured controls.

### Melanin Formation

**FIGURE 2:**
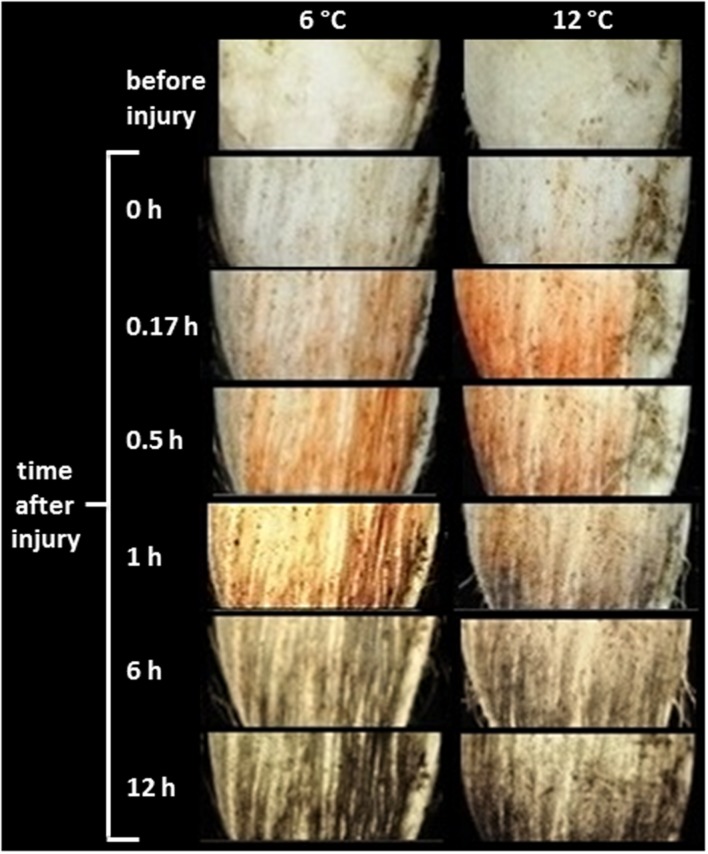
**Time course of discoloration of the wounded surface of roots stored at 6 and 12°C.** Roots were wounded by abrading the root surface with a wire brush 2 h after harvest and were subsequently stored at 94% relative humidity. Red discoloration is presumed to be due to oxidation of phenolic compounds to *o*-quinones by polyphenol oxidase. Gray to black discoloration is presumed to be due to reaction of *o*-quinones to form melanin. Pictures are representative of the coloration observed for five individual roots.

Wounding caused discoloration at the injured surface (**Figure 2**). Initially, wounded tissues developed a red discoloration, most likely due to oxidation of colorless phenolic compounds to reddish-brown *o*-quinones by PPO (Busch, 1999; Ibrahim et al., 2001). In roots stored at 12°C, red discoloration of the wounded surface was evident within 10 min (0.17 h). This discoloration was delayed in roots stored at 6°C, but was evident within 30 min (0.5 h) after wounding. The spontaneous reaction of *o*-quinones with themselves, proteins, amino acids, and phenolic compounds forms melanin, a brown to black insoluble polymer (Rouet-Mayer et al., 1990). Black discoloration, presumably due to melanin formation, was evident within 1 h in roots stored at 12°C, and within 6 h in roots stored at 6°C.

**FIGURE 3:**
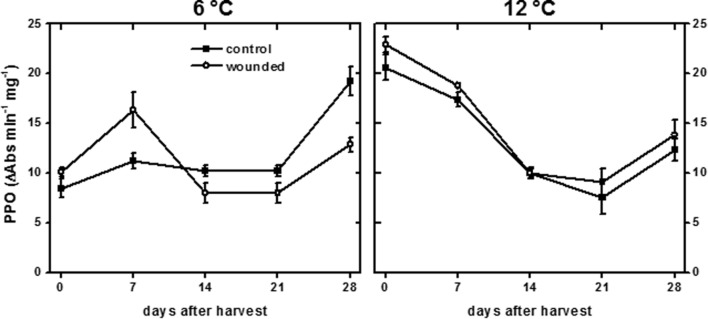
**Polyphenol oxidase (PPO) activity of wounded and unwounded (control) roots during 28 days storage at 6 and 12°C.** Roots were wounded by abrading the root surface with a wire brush 2 h after harvest and were subsequently stored at 94% relative humidity. PPO activity is expressed as ΔAbs_420_ min^-1^ (mg protein)^-1^. Data are the mean ± SE of the mean of five replicate roots.

Polyphenol oxidase activity, which was most likely responsible for producing the discoloration at the wound site, differed between roots stored at 6 and 12°C (**Figure 3**). On the day of harvest, PPO activity of roots stored at 12°C was 2.3-fold greater than that of roots stored at 6°C, and this increase at 12°C may account for the more rapid discoloration of the wound surface at 12°C than at 6°C (**Figure 2**). By 7 days, PPO activity of wounded and unwounded roots stored at 12°C declined and the activity of wounded roots at 6°C increased such that all were statistically similar. For the remainder of the 28 days storage period, PPO activity was similar between roots stored at 6 and 12°C except for an elevation in the 6°C control roots stored for 28 days. Although storage temperature had an immediate effect on PPO activity in sugarbeet roots, wounding generally had no effect on enzyme activity.

### Lignification and Suberization

**FIGURE 4:**
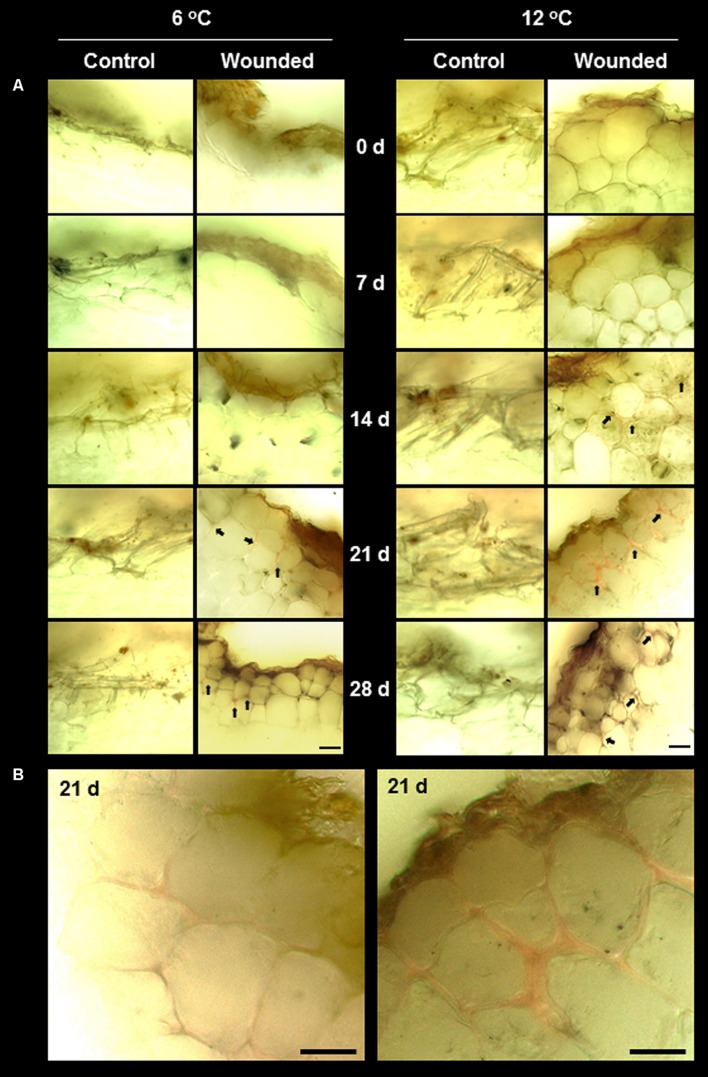
**(A)** Lignification in wounded and unwounded (control) roots stored at 6 and 12°C for up to 28 days. Lignin is stained pink–red from incubation of tissue sections in phloroglucinol prior to microscopy. Arrows identify lignin-stained areas. Roots were wounded by abrading the root surface with a wire brush after harvest and were subsequently stored at 94% relative humidity. Representative transverse sections of root surface tissues are presented. Gold–brown pigmentation at the outermost cell layers in wounded roots is believed to be melanin. Bar = 100 μm. In **(B)**, images of wounded tissue stored at 6 and 12°C for 21 days are enlarged to accentuate differences in the extent of lignification between roots stored at the two temperatures.

Lignin and suberin are commonly synthesized after wounding to seal off injuries, prevent dehydration, and prevent the ingress of pathogens (Hawkins and Boudet, 1996; Lulai and Corsini, 1998; Ramamurthy et al., 2000). In surface-abraded sugarbeet roots stored at 6 and 12°C, lignin formation at the wound site was minimal, even after 28 days (**Figure 4**). Lignin was identified by the pink–red color formed by staining with acidic phloroglucinol (Jensen, 1962). Staining indicative of lignification was first detected in wounded roots stored at 12°C after 14 days. In injured roots stored at 6°C, weak signs of lignification were first detected after 21 days. At both storage temperatures, lignification was localized to the walls of cells that were two cell layers below the wound site. By 21 days, a nearly continuous layer of lignin was evident between the second and third cell layers beneath the injury in roots stored at 12°C (**Figure 4B**). At 6°C, lignification was limited to the corners of cells, even after 28 days. No lignification was detected in the outer cell layers of unwounded controls at either storage temperature.

**FIGURE 5:**
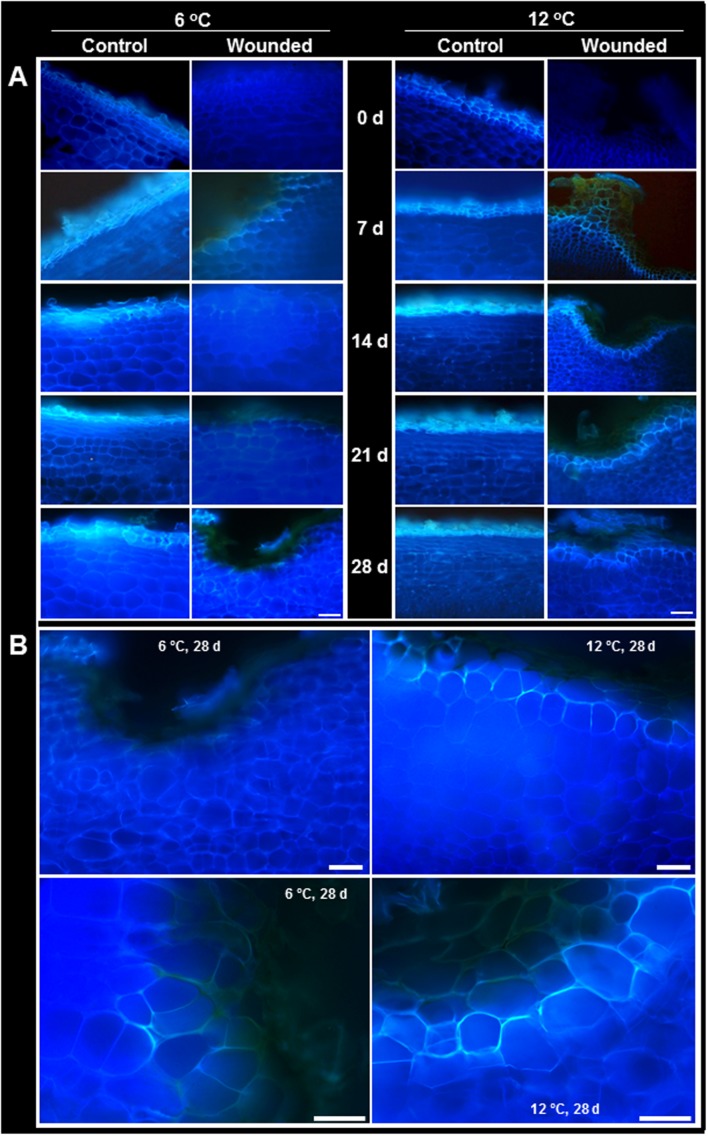
**(A)** Autofluorescence in wounded and unwounded (control) roots stored at 6 and 12°C for up to 28 days. Autofluorescence indicates the presence of suberin and lignin polyphenolic compounds. Roots were wounded by abrading the root surface with a wire brush after harvest and were subsequently stored at 94% relative humidity. Representative transverse sections of root surface tissues are presented. Gold–brown pigmentation at the outermost cell layers in wounded roots is believed to be melanin. Bar = 100 μm. In **(B)**, the autofluorescence of wounded tissue stored at 6 and 12°C for 28 days are enlarged to accentuate differences in suberization between roots stored at the two temperatures.

Suberin formation greatly exceeded lignin formation and was evident in both wounded and unwounded tissues of roots stored at 6 and 12°C (**Figure 5**). Suberization was detected by the autofluorescence of polyphenolic compounds (Lulai, 2007). Although both lignin and suberin autofluoresce, autofluorescence was due primarily to suberin since phloroglucinol staining established that lignin was undetectable in unwounded roots and present at only low levels in injured roots (**Figure 4**). Unwounded roots contained a suberized periderm comprised of several cell layers. Wounding largely removed this periderm (**Figure 5A**, 0 days). By 7 days, wound-induced suberization was evident in the cell layers beneath the injury, approximately 2–3 cell layers beneath the wound surface, in roots at both storage temperatures. However, in roots stored at 12°C, a continuous layer of suberized cells was evident by 7 days, and cells within this layer were suberized on all sides, i.e., on outer tangential, inner tangential, and radial walls. At 6°C, the perimeters of cells were not completely suberized, and a continuous layer of suberized cells was not formed, even after 28 days in storage.

Lignin and suberin biosyntheses require the activity of PAL and POD. PAL catalyzes the transformation of phenylalanine to *trans*-cinnamic acid and controls entry of carbon compounds into the phenylpropanoid pathway (Hahlbrock and Scheel, 1989). The phenylpropanoid pathway generates the monolignols and phenolic compounds that serve as substrates for lignin and suberin biosynthesis. Peroxidase is generally believed to be responsible for polymerizing monolignols and phenolic compounds from the phenylpropanoid pathway into lignin and suberin polymers (Quiroga et al., 2000).

**FIGURE 6:**
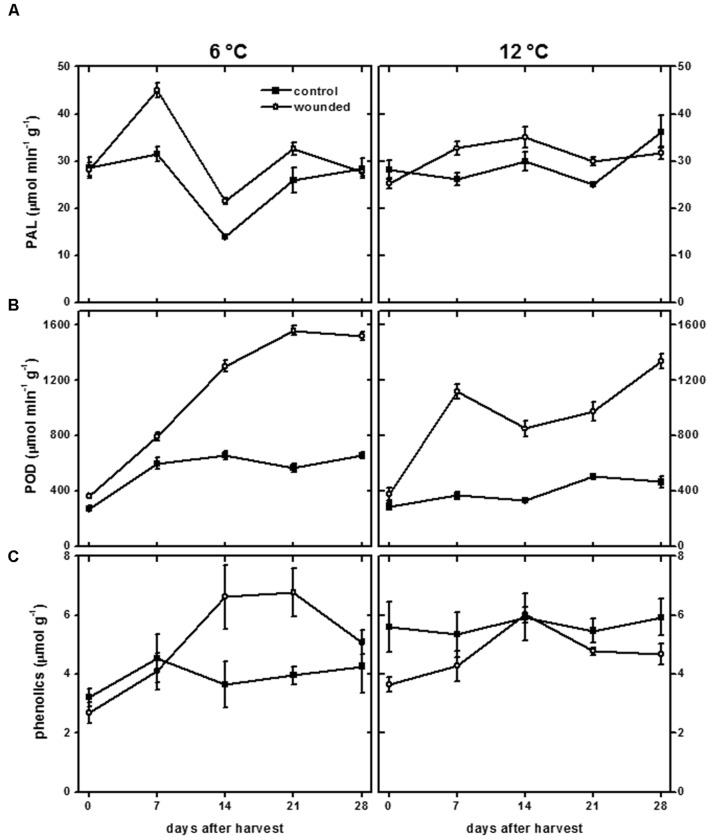
**Phenylalanine ammonia lyase (PAL) activity (A), peroxidase (POD) activity (B), and total phenolics concentration (C) of wounded and unwounded (control) roots during 28 days storage at 6 and 12°C.** Roots were wounded by abrading the root surface with a wire brush 2 h after harvest and were subsequently stored at 94% relative humidity. PAL and POD activity are expressed as μmol product min^-1^ (g protein)^-1^. Total phenolics are expressed as μmol gallic acid equivalents (g dry wt)^-1^. Data are the mean ± SE of the mean of five replicate roots.

Wounding had a small effect on PAL activity and a large effect on POD activity in roots stored at 6 and 12°C (**Figure 6**). At both storage temperatures, PAL activity exhibited small and transient increases due to wounding (**Figure 6A**). At 12°C, PAL activity was 20–25% greater in wounded roots than in control roots at 7 and 21 days after harvest. At 6°C, PAL activity was 43–53% greater in wounded roots compared to controls at 7 and 14 days after harvest. POD activity was elevated in wounded roots throughout the 28 days storage period at both temperatures (**Figure 6B**). The increase in POD activity due to injury occurred more rapidly at 12°C than at 6°C. After 7 days storage, POD activity in injured roots was elevated 2.8-fold at 12°C and 1.9-fold at 6°C, relative to control roots on the day of harvest. However, the increase in POD activity was greatest in roots stored at 6°C. After 28 days in storage, POD activity of wounded roots increased 4.5-fold at 6°C and 3.6-fold at 12°C, relative to control roots on the day of harvest. PAL and POD activities were unrelated to lignification (**Figure 4**) and suberization (**Figure 5**) in wounded sugarbeet roots. Overall, PAL and POD activities were elevated to a greater extent at 6°C than at 12°C in wounded roots, while suberization and lignification were greater at 12°C than at 6°C.

The concentration of total phenolic compounds is determined by the rates of both their biosynthesis and their utilization (Becerra-Moreno et al., 2015). In wounded tissue, the concentration of total phenolic compounds was significantly reduced in roots stored at 12°C and significantly elevated in roots stored at 6°C (**Figure 6C**). Over the 28 days of the experiment, the concentration of total phenolic compounds was reduced 17% in wounded roots stored at 12°C relative to unwounded controls, consistent with an increase in their utilization, possibly for lignin and suberin formation (**Figures 4** and **5**), without a concomitant increase in their synthesis, as reflected by the minimal changes in PAL activity at 12°C (**Figure 6A**). At 6°C, the concentration of total phenolic compounds was 29% greater in wounded roots relative to controls over the 28 days of the experiment. This increase may reflect increased synthesis of phenolic compounds due to increased PAL activity (**Figure 6A**), and limited use of phenolic compounds for lignin and suberin formation (**Figures 4** and **5**).

### Respiration

**FIGURE 7:**
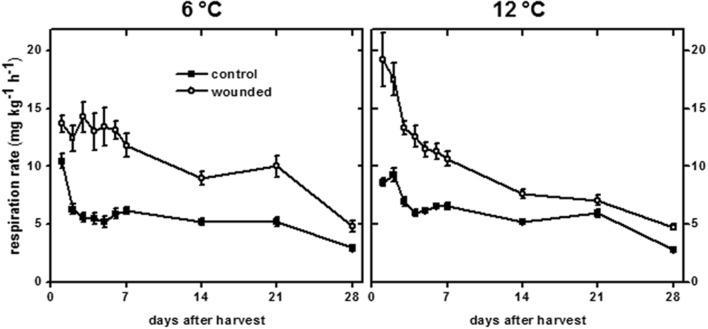
**Respiration rate of wounded and unwounded (control) roots during 28 days storage at 6 and 12°C.** Respiration rate is expressed as mg CO_2_ kg^-1^ h^-1^. Roots were wounded by abrading the root surface with a wire brush 2 h after harvest. Roots were stored at 94% relative humidity. Data are the mean ± SE of the mean of eight replicate roots.

Wounding typically induces large increases in respiration in harvested plant products (Kays and Paull, 2004; Lafta and Fugate, 2011) and occurs, presumably, to provide metabolic energy and substrates to support wound-healing processes (Lipetz, 1970). For sugarbeet roots stored at both 6 and 12°C, wounding significantly increased respiration rate, although the intensity and duration of wound-induced respiration differed between the two temperature treatments (**Figure 7**). The initial increase in respiration due to wounding was greatest for roots stored at 12°C. After 1 days storage, wounded roots stored at 12°C respired at a rate that was 40% greater than that of wounded roots stored at 6°C. The respiration rate of wounded roots at 12°C rapidly declined with time in storage. By the 3rd day in storage, the decline in respiration rate of wounded roots was significant, and respiration rate was not significantly different from unwounded controls after 21 days in storage. At 6°C, the respiration rate of wounded roots remained elevated for the first 7 days in storage at an average rate of 13.1 mg CO_2_ kg^-1^ h^-1^. Respiration rate declined after 14–21 days in storage to an average of 9.5 mg CO_2_ kg^-1^ h^-1^, and did not decline to levels similar to control roots until 28 days after wounding. Although the initial increase in respiration rate due to wounding was greater in roots stored at 12°C, integration of the area between the curves for wounded and control roots at 6 and 12°C indicated that the increase in respiration due to wounding over 28 days in storage was 52% greater in roots stored at 6°C than in roots stored at 12°C.

Changes in respiration rate due to wounding and storage temperature (**Figure 7**) mirrored changes in transpiration rate (**Figure 1A**). Using data for both wounded and unwounded roots stored at 6 and 12°C, a correlation coefficient of 0.765 (*P* < 0.001) was found for the relationship between respiration rate and transpiration rate. Since transpiration rate is a measure of wound-healing, this relationship suggests that respiration rate is related to injury and the extent to which that injury has healed.

### Antioxidant Defenses

**FIGURE 8:**
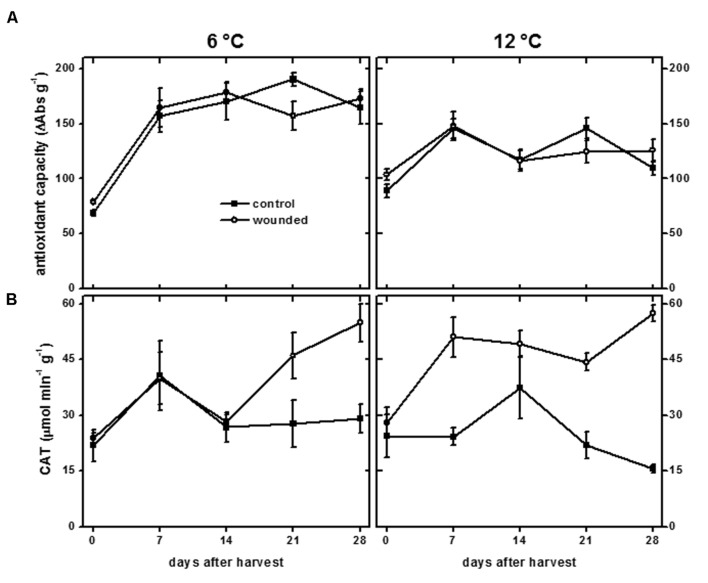
**Antioxidant capacity (A) and catalase (CAT) activity (B) of wounded and unwounded (control) roots during 28 days storage at 6 and 12°C.** Roots were wounded by abrading the root surface with a wire brush 2 h after harvest. Roots were stored at 94% relative humidity. Antioxidant capacity is expressed as ΔAbs_517_ (g dry wt)^-1^. CAT activity is expressed as μmol H_2_O_2_ destroyed min^-1^ (g protein)^-1^. Data are the mean ± SE of the mean of five replicate roots.

Induction of antioxidant defenses has been observed in many plant species in response to wounding, including increases in antioxidant capacity and CAT activity (Kang and Saltveit, 2002; Reyes and Cisneros-Zevallos, 2003; An et al., 2009). Antioxidant capacity is a measure of the capacity of soluble cellular components such as phenolic compounds, ascorbic acid, tocopherol, and flavonoids, to scavenge free radicals (Brand-Williams et al., 1995). In sugarbeet roots, antioxidant capacity was unaffected by wounding at either storage temperature (**Figure 8A**). Antioxidant capacity, however, increased during storage at 6 and 12°C. At 6°C, antioxidant capacity increased 130%. At 12°C, antioxidant capacity increased 30%. Although phenolic compounds contribute to antioxidant capacity, no correlation was observed between antioxidant capacity (**Figure 8A**) and the concentration of phenolic compounds (**Figure 6C**).

Catalase catalyzes the conversion of H_2_O_2_ to H_2_O and O_2_ and is involved in antioxidant defenses and H_2_O_2_ metabolism. In addition, CAT activity has been found to be positively correlated with wound-healing efficiency, at least in potato, where its activity was required for deposition of the polyphenolic domain of suberin (Bajji et al., 2007). In sugarbeet roots, wounding increased CAT activity, with wound induction of CAT activity occurring more rapidly in roots stored at 12°C than at 6 °C (**Figure 8B**). The increase in CAT activity due to injury was statistically significant after 7 days at 12°C, coincident with suberization beneath the site of injury (**Figure 5**). In contrast, CAT activity in roots stored at 6°C was not significantly elevated in wounded roots relative to unwounded controls until 28 days after harvest. CAT activity in wounded roots at 6°C was unrelated to suberization which was evident 7 days after injury (**Figure 5**).

## Discussion

Since unhealed wounds facilitate water vapor loss and pathogen infection, promote root dehydration, increase the prevalence of storage diseases, and accelerate sucrose loss and the deterioration of root quality during storage, healing of wounds incurred during harvest and piling operations is critical for successful long-term storage of sugarbeet roots (Campbell and Klotz, 2006; Lafta and Fugate, 2009). For a variety of root and tuber crops including potato, sweet potato, yam, cassava, and carrot, it is well established that wound healing is affected by storage temperature (Nielsen and Johnson, 1974; Wiggington, 1974; Booth, 1976; Garrod et al., 1982; Diop, 1998). The effect of storage temperature on sugarbeet root wound healing, however, is largely unknown. To begin to understand the interaction of storage temperature and wound healing in sugarbeet roots, research was conducted to determine the effect of temperature on a number of wound-healing and wound-related processes including root transpiration rate, weight loss, suberization, lignification, melanin formation, respiration rate, and activities of enzymes involved in wound-healing processes, using sugarbeet roots that were surface-abraded and stored at 6 and 12°C for 28 days. From this research, we provide evidence that sugarbeet root wound-healing processes are impacted by storage temperature and conclude that cold temperature impairs both the rate and extent of wound healing in stored sugarbeet roots.

Wound healing was clearly evident in sugarbeet roots stored at 12°C. Declines in transpiration rate from the wounded surface (**Figure 1A**) and the lack of acceleration in weight loss in wounded roots during storage relative to controls (**Figure 1B**) indicated that wounded roots at 12°C were able to seal off wound sites and restrict water vapor loss. Suberin formation was likely involved in creating a barrier against water loss, as wounded roots stored at 12°C developed a continuous layer of suberized cells, approximately 2–3 cell layers beneath the wound site, within 7 days after injury (**Figure 5**). Suberin deposition is a common component of wound healing in other root and tuber crops (Nielsen and Johnson, 1974; Garrod et al., 1982; Lulai and Corsini, 1998). A polymer with polyphenolic and polyaliphatic domains that is deposited between the primary cell wall and the plasma membrane (Bernards, 2002), suberin creates a water-impermeable barrier that has been demonstrated to restrict both water loss and pathogen ingress in other wounded plant products (Lulai and Orr, 1995; Lulai and Corsini, 1998).

In contrast, wound healing in roots stored at 6°C was considerably delayed and impaired. In these roots, transpiration rate exhibited no significant declines during 28 days in storage (**Figure 1A**) and weight loss in storage was accelerated relative to unwounded roots (**Figure 1B**), indicating that wounded roots at 6°C were unable to seal off wound sites and restrict water vapor loss, even after 28 days. Suberization was detected in wounded roots stored at 6°C (**Figure 5**). However, suberin biosynthesis at 6°C was severely delayed or impaired relative to roots stored at 12°C. At 12°C, suberized cells beneath the wound site were suberized over the entire circumference of the cell. At 6°C, cell perimeters of suberized cells beneath the wound site were incompletely suberized, and suberized cells did not form a continuous suberized layer beneath the wound site during 28 days in storage. Whether wounded roots at 6°C would seal off wounds with additional storage time is unknown. However, in other root and tuber crops such as potato, sweet potato and carrot, low temperature delays, but does not prevent, formation of suberized barriers (Nielsen and Johnson, 1974; Wiggington, 1974; Garrod et al., 1982).

Lignin biosynthesis was detected in surface-abraded sugarbeet roots stored at 6 and 12°C (**Figure 4**). Lignin, a polymer comprised primarily of the monolignols, *p*-coumaryl, coniferyl, and sinapyl alcohols, is often produced after wounding and is believed to contribute to wound healing by creating an impenetrable barrier to water and pathogens (Hawkins and Boudet, 1996; Rogers and Campbell, 2004). In surface-abraded sugarbeet roots, lignin biosynthesis occurred after suberin formation and was first detected 14 and 21 days after wounding in roots stored at 12 and 6°C, respectively. Lignin formation was greater in wounded roots stored at 12°C than at 6°C. Nevertheless, very little lignin was detected at either storage temperature, suggesting that lignin biosynthesis is not a major contributor to wound healing in surface abraded sugarbeet roots.

Production of lignin and suberin in response to injury differs between plant species, organs, tissues, and injury types in regards to the rate of formation, the order of appearance of lignin and suberin, and their relative quantities (Artschwager, 1924, 1927; Garrod et al., 1982; Rittinger et al., 1987; Hawkins and Boudet, 1996). The pattern of lignification and suberization observed for surface-abraded sugarbeet roots was similar to that observed for wounded carrot roots and potato tubers (Garrod et al., 1982; Lulai, 2007). In these systems, suberization precedes lignification, and suberin is largely responsible for sealing off the wound site. In contrast, Ibrahim et al. (2001) reported that lignification preceded and exceeded suberin formation during wound healing of bruised sugarbeet roots stored at 10°C. The differences in lignin and suberin biosynthesis between surface-abraded and bruised sugarbeets is unknown but may be due to differences in the type of injury sustained. In potato, wound-healing processes differ between tubers that sustain cut or stab wounds and tubers that are bruised (Artschwager, 1924, 1927).

Correlations between lignin or suberin formation and PAL or peroxidase activities have been found in other plant species and organs, suggesting that these enzymes participate in the regulation of lignin and suberin biosynthesis (Lee et al., 1992; Quiroga et al., 2000; Kao et al., 2002; Xue et al., 2008). In wounded sugarbeet roots, however, PAL and POD activities (**Figures 6A,B**) had no relationship to lignification (**Figure 4**) or suberization (**Figure 5**). Alterations in PAL and POD activities, therefore, were unlikely to be responsible for the differences in lignin and suberin formation that occurred in surface-abraded roots stored at 6 and 12°C. CAT activity, which has been implicated in suberin formation in potato (Bajji et al., 2007), increased in response to wounding (**Figure 8B**). This increase, however, was unrelated to suberin formation (**Figure 5**). Similarly, no evidence was found to indicate that the availability of phenolic substrates contributed to the regulation of lignin and suberin biosynthesis in surface-abraded sugarbeet roots. Generally, the concentration of total phenolic compounds in wounded roots was elevated at 6°C and reduced at 12°C (**Figure 6C**), while lignification and suberization were greater in wounded roots stored at 12°C than at 6°C (**Figures 4** and **5**). Although wound-induced changes in phenolics have been associated with wound-induced changes in antioxidant capacity in other plant products (Reyes et al., 2007), the concentration of total phenolic compounds was not related to antioxidant capacity (**Figure 8A**) in surface-abraded sugarbeet roots. However, the absence of correlation between total phenolic compounds and lignin biosynthesis, suberin biosynthesis or antioxidant capacity does not preclude the possibility that concentration changes of individual phenolic compounds may relate to lignin or suberin formation or be responsible for changes in antioxidant capacity. In carrot roots and potato tubers, individual phenolic compounds differed in their response to wounding, and alterations in the concentrations of individual phenolic compounds occurred even when the concentration of total phenolics was relatively unchanged (Torres-Contreras et al., 2014a,b; Becerra-Moreno et al., 2015). Moreover, changes in antioxidant capacity due to changes in the profile of phenolic compounds have been documented when no significant relationships between antioxidant capacity and the concentration of total phenolic compounds exist (Jacobo-Velázquez and Cisneros-Zevallos, 2009).

Melanin formation was observed in surface-abraded roots stored at both 6 and 12°C (**Figure 2**). Melanin formed more rapidly at 12°C than at 6°C, although roots at both temperatures exhibited similar levels of melanin within 6 h after injury. Since roots stored at 6 and 12°C differed in their ability to seal off wounds (**Figure 1**) but had similar levels of melanin within the 1st day after injury (**Figure 2**), melanin was not involved in sealing wound sites or restricting water vapor loss in surface-abraded sugarbeet roots. The function of melanin formation in surface-abraded sugarbeet roots is unknown. Roles in cell wall strengthening and defense against microbial infections, however, have been proposed in other plant systems (Riley, 1997; Wiltshire and Cobb, 2000). Melanin biosynthesis is catalyzed by PPO (Brown et al., 1999). In surface-abraded sugarbeet roots, PPO activity was more than twofold higher at 12°C than at 6°C at 30 min after wounding (**Figure 3**). Differences in PPO activity at 6 and 12°C may account for the rapidity of melanin biosynthesis at 12°C relative to its biosynthesis at 6°C. Similar to this study, positive correlations between PPO activity and storage temperature have been found in other commodities (Mondy et al., 1966; Concellón et al., 2004).

Storage temperature affected wound-induced respiration throughout the 28 days storage period (**Figure 7**). Wound-induced increases in respiration rate were initially higher for wounded roots stored at 12°C than for wounded roots stored at 6°C. However, respiration rates of wounded roots declined more rapidly as a function of time in storage at 12°C than at 6°C. As a result, the wound-induced elevation in respiration rate over 28 days was greater in roots stored at 6°C than at 12°C. Wound-induced respiration (**Figure 7**) was positively correlated with transpiration rate, a measure of wound-healing (**Figure 1A**). The overall reduction in respiration rate at 12°C relative to that at 6°C, therefore, was likely due to greater and more rapid wound-healing at 12°C. In sugarbeet roots, sucrose is the primary substrate for respiration, and respiration is the primary cause of sucrose loss in storage (Barbour and Wang, 1961; Wyse and Dexter, 1971). Rapid healing of wounds, therefore, is likely to reduce wound-induced respiration and decrease sucrose loss in storage.

Comparison of surface transpiration rate, root weight loss, suberization, lignification, melanin formation, respiration rate, and activities of enzymes involved in wound-healing processes of surface-abraded sugarbeet roots stored at 6 and 12°C generated clear evidence that wound healing in harvested sugarbeet roots is affected by storage temperature. All parameters of wound healing that were investigated in this study were affected by storage temperature, and by all measures, wound healing was more rapid and complete in roots stored at 12°C than at 6°C. From these results, we conclude that cold temperature impairs the rate and extent of wound healing of stored sugarbeet roots. Current practice in the sugarbeet industry is to cool harvested roots as quickly as possible to reduce root respiration rate and slow growth of pathogens that cause storage diseases (Campbell and Klotz, 2006; Fugate and Campbell, 2009). This research indicates, however, that if freshly harvested roots are cooled too rapidly, wound healing will be delayed, and with these delays, storage losses are likely to be exacerbated due to wound-related elevations in respiration rate, storage disease, and dehydration (Mumford and Wyse, 1976; Campbell and Klotz, 2006; Lafta and Fugate, 2009).

## Author Contributions

KF, FF, and WR conceived and planned the study. KF and FF drafted the manuscript. FF and WR carried out experiments. EL and ED provided technical assistance and assisted with data analysis and manuscript revision.

## Conflict of Interest Statement

The authors declare that the research was conducted in the absence of any commercial or financial relationships that could be construed as a potential conflict of interest.
